# Supplementary material for: Differential Effects of Somatostatin, Octreotide, and Lanreotide on Neuroendocrine Differentiation and Proliferation in Established and Primary NET Cell Lines: Possible Crosstalk with TGF-β Signaling
Source: Int J Mol Sci. 2022 Dec 14;23(24):15868. doi: 10.3390/ijms232415868 (PMC9781720; doi:10.3390/ijms232415868)
Supplement: Supplementary file 1 [file ijms-23-15868-s001.zip › ijms-2023943-supplementary.pdf]

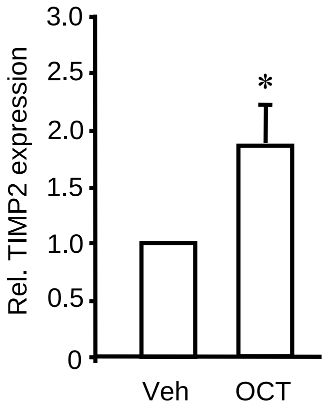

Figure S1. Upregulation of TIMP2 expression in OCT-treated BON cells. BON cells were challenged with either vehicle (Veh) or OCT (1 μM) for 24 h, followed by RNA isolation and qPCR analysis. The data shown are the means ± SD from three assays ($n = 3$). Veh control cells are set arbitrarily at 1.0. The asterisk (∗) denotes a significant difference ($p < 0.05$, Wilcoxon test).
